# Role of mast cells in the generation of a T-helper type 2 dominated anti-helminthic immune response

**DOI:** 10.1042/BSR20181771

**Published:** 2019-02-15

**Authors:** Nathan M. Ryan, Steve Oghumu

**Affiliations:** Department of Pathology, College of Medicine, Ohio State University Wexner Medical Center, Columbus, OH, U.S.A.

**Keywords:** Helminth, immune response, Mast cell

## Abstract

Mast cells are long-lived, innate immune cells of the myeloid lineage which are found in peripheral tissues located throughout the body, and positioned at the interface between the host and the environment. Mast cells are found in high concentrations during helminth infection. Using *Kit^w-sh^* mast cell deficient mice, a recently published study in *Bioscience Reports* by Gonzalez et al. (Biosci. Rep., 2018) focused on the role of mast cells in the immune response to infection by the helminth *Hymenolepis diminuta*. The authors showed that mast cells play a role in the modulation of Th2 immune response characterized by a unique IL-4, IL-5 and IL-13 cytokine profile, as well as subsequent robust worm expulsion during *H. diminuta* infection. Unlike WT mice which expelled *H. diminuta* at day 10, *Kit^w-sh^* deficient mice displayed delayed worm expulsion (day 14 post infection). Further, a possible role for mast cells in the basal expression of cytokines IL-25, IL-33 and thymic stromal lymphopoietin was described. Deletion of neutrophils in *Kit^w-sh^* deficient mice enhanced *H. diminuta* expulsion, which was accompanied by splenomegaly. However, interactions between mast cells and other innate and adaptive immune cells during helminth infections are yet to be fully clarified. We conclude that the elucidation of mechanisms underlying mast cell interactions with cells of the innate and adaptive immune system during infection by helminths can potentially uncover novel therapeutic applications against inflammatory, autoimmune and neoplastic diseases.

Mast cells (MC) are long-lived, granulated, tissue resident effector cells of hematopoietic origin, recognized for their role in allergic inflammation and immunity to parasitic infection. They derive from common myeloid progenitors in the bone marrow, and continue their development through the granulocyte/monocyte progenitor lineage. Development into MC progenitors in the bone marrow is highly regulated by transcription factors. Cells committed to the MC lineage leave the bone marrow as MC progenitors, and circulate in the bloodstream before homing to peripheral tissues including the skin, lung, peritoneum and the intestinal epithelia [1]. Two major subsets have been identified: connective tissue MCs and mucosal MCs. Mast cell development and survival are dependent on the surface expression of the receptor tyrosine kinase c-kit, present in the W-locus (chromosome 5) in mice. C-kit is a receptor for the ligand, stem cell factor (SCF), an important growth factor for MC. Indeed, mutations at the c-kit locus have been used extensively for the study of MC deficiency in mice. For example, mice with the *W-sh* mutation (*Kit^W-sh/W-sh^*) which possess an inversion mutation in the transcriptional regulatory region of the *c-kit* gene [2] results in a significant reduction in *c-kit* mRNA and subsequently MC deficiency in peripheral tissues. In addition to mutant Kit-based mast cell deficient mice, (*Kit^W-sh/W-sh^* and WBB6F1-*KitW/Wv*), genetically modified mouse models such as *Mcpt5Cre* and *Cpa3Cre* have been shown to be useful in addressing the function of MC *in vivo* [3].

MCs are known mediators of anti-helminthic responses such as infections with *Heligmosomoides polygyrus, Trichuris suis, Schistosoma japonicum, Necator americanus, Strongyloides venezuelensis, Trichinella spiralis* and *Trichuris muris* [4–11] (Table 1). In the context of helminthic infection, the effector functions of MC are largely mediated by high affinity interactions of the IgE receptor, FcεR1 present on MC. Interaction between IgE and FcεR1 results in the activation and subsequent release of cytosolic granules by MC. These granules contain a number of cytokines, growth factors, and proteases including interleukin (IL)-4, IL-5, vascular endothelial growth factor (VEGF), tumor necrosis factor (TNF) and mast cell protease 1 (MCPT-1), which can be detected as free MCPT-1 in the serum or tissues as an indicator for the presence of MC *in vivo* [12]. Helminthic infections trigger a number of host responses, largely characterized by a Th2 polarized immune response. In response to helminth infection, innate immune cells and intestinal epithelial cells secrete Th2 cytokines including IL-4, IL-5, IL-9, IL-13, IL-25, IL-33 and thymic stromal lymphopoietin (TSLP) [13]. IL-33, a cytokine released during helminth infection, causes the activation and proliferation of MC through interaction with the ST2 receptor [14,15]. Activation of MC results in their degranulation and release of pre-formed mediators known to modulate cells of the innate and adaptive immune system. Among these are IL-4 and IL-13 resulting in the alternative activation of macrophages [16], prostaglandin D2, which cleaves IL-33 resulting in increased type 2 innate lymphoid cell induction through CRTH2 receptor interaction [17], and TNF-α, CXCL1 and CXCL2 leading to the recruitment and proliferation of neutrophils at the site of infection [18,19]. TNF-α mediated neutrophil recruitment by MC has further been shown to be at least partially dependent MC activation by IL-33 [19]. In addition to the activation and recruitment of cells of the innate and adaptive immune system, MC degranulation induces effector mechanisms involved in worm expulsion such as goblet cell hyperplasia, increased mucin production, mitigation of tissue damage, intestinal smooth muscle contraction associated with heightened peristalsis, and the creation of an environment toxic to helminths [20]. Although MCs are known mediators of the helminth associated Th2 response, it is evident that their roles vary, depending on the host, parasite dose, parasite life cycle stage, and duration of infection [21].

**Table 1:** Helminthic infections: role of mast cells and suppression of autoimmune inflammatory diseases

Helminth	MC involvement in helminth infection	Amelioration of inflammatory disease	
		Disease	Model
*Hymenolepis diminuta* (Rat Tapeworm)	MC contributes to helminth expulsion	DNBS/DSS colitis [22,23,44–47]	Experimental (Mouse)
	[24,43]	Autism [48]	Clinical
		Arthritis [49]	Experimental (Mouse)
*Trichuris suis* (Pig Whipworm)	MC accumulates during infection [9]	DSS colitis [50]	Experimental (Rabbit)
		EAE [51]	Experimental
		OVA-sensitization [52]	Experimental (Mouse)
		Ulcerative colitis [53,54]	Clinical trial
		Crohn’s disease [54–56]	Clinical trial
		Multiple sclerosis [57]	Clinical trial
		Allergic rhinitis [58]	Clinical trial
		Peanut/Treenut allergy [59]	Clinical trial
		Plaque psoriasis [60]	Clinical trial
		Autism [48,61]	Clinical trial
*Necator americanus* (Human Hookworm)	MC accumulation and degranulation	Crohn’s disease [62]	Clinical trial
	correlate with protection against helminth	Celiac disease [63]	Clinical trial
	[10]	Asthma [64–66]	Clinical trial
		Multiple sclerosis [67]	Clinical trial
		Allergic rhinitis [68]	Clinical trial
*Trichuris trichuria* (Human Whipworm)	Not studied	Ulcerative colitis [69]	Clinical trial
		Atopic dermatitis [70]	Clinical trial
		Multiple sclerosis [71]	Clinical trial
*Schistosoma mansoni*	Conflicting data; most evidence suggest	EAE [76,77]	Experimental (Mouse)
	that MC accumulation correlates with	NOD [78,79]	Experimental (Mouse)
	susceptibility to infection [72–75]	TNBS/DSS colitis [80–82]	Experimental (Mouse, Rat)
		OVA-sensitization [83,84]	Experimental (Mouse)
		Anaphylaxis [85]	Experimental (Mouse)
		TSHR (Graves’ disease) [86]	Experimental (Mouse)
		CIA [87]	Experimental (Mouse)
*Trichinella spiralis*	Apparent involvement of MC in helminth	EAE [90–92]	Experimental (Rat)
	expulsion [88,89]	NOD [93]	Experimental (Mouse)
		DNBS colitis [94–97]	Experimental (Mouse)
*Heligmosomoides polygyrus*	MC play a major role in clearance of	EAE [100]	Experimental (Mouse)
	infection [98,99]	NOD [93]	Experimental (Mouse)
		IBD [101–103]	Experimental (Mouse)
		OVA-sensitization [100,104,105]	Experimental (Mouse)
		Arthritis [106]	Experimental (Mouse)
		Peanut allergy [107]	Experimental (Mouse)
		TNBS colitis [108,109]	Experimental (Mouse)
*Trichinella pseudospiralis*	MC accumulates during infection [110]	EAE [111]	Experimental (Mouse)
*Taenia crassiceps*	MC accumulates during infection [112]	MLDS [113]	Experimental (Mouse)
		EAE [114]	Experimental (Mouse)
		DSS colitis [115]	Experimental (Mouse)
*Litomosoides sigmodontis*	MC degranulation promotes helminth	NOD [118,119]	Experimental (Mouse)
	invasion and survival in host [116,117]	OVA-sensitization [120]	Experimental (Mouse)
		DIO [121]	Experimental (Mouse)
*Ancylostoma caninum*	MC accumulates during infection [122]	DSS colitis [123]	Experimental (Mouse)
*Strongyloides venezuelensis*	MC play a major role in clearance of infection [124,125]	MLDS [126]	Experimental (Mouse)
*Nippostrongylus brasiliensis*	MC contributes to helminth expulsion	OVA-sensitization [128]	Experimental (Mouse)
	[127]	Arthritis [106]	Experimental (Mouse)
*Schistosoma japonicum*	MC accumulates during infection [11]	OVA-sensitization [129,130]	Experimental (Mouse)
		TNBS colitis [131]	Experimental (Mouse)
		EAE [132]	Experimental (Mouse)
*Trichuris muris*	MC accumulates but not required for	DSS colitis [136]	Experimental (Mouse)
	protection against infection [133–135]	AAI [137]	Experimental (Mouse)

Infection of the rat tapeworm *Hymenolepis diminuta* in mice is an established model system used to elucidate the complex immune response mechanisms to chronic intestinal helminthic infections in humans. Because these tapeworms possess potent immunosuppressive properties during concomitant inflammatory disease states (such as colitis), and are known to cause minimal to no tissue damage within the host they are ideal models for the study of helminth-associated immunological responses [22]. Not surprisingly, the immunomodulatory and anti-inflammatory properties of *H. diminuta* are potentially being exploited in the treatment of gut-associated inflammatory diseases – an area currently known as ‘helminth therapy’ which is currently under active investigation [23].

Given our limited understanding of the specific roles of MC during infection with *H. diminuta*, the study by González et al. [24] begins to define the immunomodulatory function of MC against this helminth *in vivo.* Mice are known to generate a strong Th2 polarized immune response against *H. diminuta* and clear infection in 8–10 days [25]. Using C57BL/6 mice with *Kit^W-sh/W-sh^* mutations, which depletes MC [26], a revealing picture is beginning to emerge suggesting a role for MC in effective elimination of *H. diminuta* from infected hosts. Previous studies involving infection by *H. diminuta* have followed MC activity using rat models, which generate a wide array of MC activation profiles dependent upon helminth dose and rat species, making the elucidation of MC effects on elimination of helminths from the gut lumen challenging [27]. In mice, activation of MC in response to *H. diminuta* infection has been demonstrated as indicated by the detection of MCPT-1, a MC biomarker detectable in serum [28]. *Kit^W-sh/W-sh^* mice lacking in a MC response as shown through the non-detectable levels of serum MCPT-1 have been used previously to demonstrate the wide ranging effects that MC can have in mediating an effective Th2 polarized anti-helminthic immune response *in vivo* [29]. During *H. diminuta* infection, *Kit^W-sh/W-sh^* mice produced an altered Th2 cytokine immune response profile, which differed in kinetics compared with infected WT mice. Interestingly, infected *Kit^W-sh/W-sh^* mice produced higher quantities of IL-4 and IL-13 at day 4, but lower levels at day 8 compared with infected WT controls. Levels of these cytokines are again reversed at day 12, with an apparent rebound in IL-4 and IL-13 production in infected *Kit^W-sh/W-sh^* mice. While this altered Th2 cytokine profile in infected *Kit^W-sh/W-sh^* mice appears to be MC dependent, additional studies are needed to determine the mechanisms underlying the unique kinetics of Th2 cytokine production during *H. diminuta* infection of *Kit^W-sh/W-sh^* mice. Nevertheless, these results suggest a role for activated MC in modulating Th2 cytokine production during *H. diminuta* infection, [30]. It must be noted, however, that while non-detectable levels of MCPT-1 are strongly indicative of complete absence of MC (which was used as a surrogate for MC in the study by Gonzalez et al. [24]), there have been demonstrated instances of a MC presence occurring in *Kit*-deficient animals [31,32]. Nevertheless, the delayed worm expulsion seen experimentally does demonstrate an as yet unknown role for MC in the optimal generation of an effective immune response against *H. diminuta*.

In addition to the aforementioned cytokines (IL-4, IL-5 and IL-13), González et al. [24] found that basal expression of epithelia derived cytokines IL-25, IL-33, and TSLP in uninfected *Kit^W-sh/W-sh^* deficient mice was lower compared with uninfected WT controls. This suggests a role for MC maintaining homeostatic basal expression for these cytokines. Previous research performed using different helminth models demonstrates a role for MC in the production of IL-25, IL-33, and TSLP, suggesting the possibility of MC priming of these cytokines during early infection [4]. In *H. diminuta* infected mice, expression values were similar for these cytokines, indicating that while MC may assist in the maintenance of their basal expression, these cytokines can still be induced independent of MC during this helminth infection. While a clearer picture of the regulation of these epithelium derived cytokines is beginning to emerge, the precise immunologic mechanisms that underlie their production and regulation are still incompletely understood [33]. Increased expression of these cytokines has been linked to the allergic and asthma response [34], and a mechanism decreasing basal IL-25, IL-33 and TSLP expression may be of interest in potential therapy development. Importantly, these cytokines have each recently been attributed to having an important initial role in inducing a microenvironment suitable for Th2 polarization [35]. This correlation between reductions in these epithelial derived cytokines and the delayed Th2 response seen experimentally is further evidence in support of the Th2 polarizing effect of IL-25, IL-33 and TSLP in certain helminthic infections. Further research into the immunomodulatory roles and regulatory mechanisms of these epithelial derived cytokines, as well as their cross-talk with MCs during inflammation will provide insights into therapeutic approaches in the management of helminthic, gut-inflammatory and allergic diseases.

A clear understanding of the interaction between MCs and other innate and adaptive immune cells during helminthic infection is vitally important in order to clarify the nature of an effective anti-helminthic immune response. For example, the proportion of neutrophils was observed to be increased in the spleens of MC deficient *H. diminuta* infected mice. However, contrary to the notion that neutrophils could compensate for the lack of MC to confer protection against *H. diminuta* infection, Gonzalez et al. [24] found that depletion of neutrophils by intraperitoneal administration of anti-Gr-1 antibodies resulted in enhanced clearance of the worm at a rate comparable to infected WT mice. Furthermore, neutrophil depletion in MC deficient *H. diminuta* infected mice was accompanied by increased splenomegaly. These data present very interesting observations that warrant further study. While it has been previously shown that *in vivo* depletion of neutrophils can result in a more strongly polarized Th2 response in the context of helminth infection, the mechanisms that underlie this immunological response are not completely understood [36]. Further, in contrast with the data presented by Gonzalez et al. [24], neutrophil depletion in mice during *Nippostrongylus brasiliensis* helminth infection resulted in a decreased Th2 response and an increased susceptibility to infection [37]. These observations further support the wide variety and complexity of cellular interactions and immunological responses elicited by different helminth infections *in vivo.* As is the case with other parasitic diseases, infections with helminths require a consideration of pathogen and host associated factors in order to fully explain mechanisms of action and host immune response pathways. In the context of MC inhibition, it would be of interest to determine the impact of neutrophil depletion on immune response to other helminth infections.

As mentioned at the outset, the concept of helminth-based therapy exploits the immunosuppressive properties of helminth species to reduce the severity of gut associated inflammatory diseases. A summary of pre-clinical and clinical studies utilizing helminth parasites to mitigate inflammatory associated diseases is shown in Table 1. In the context of helminth infection by *H. diminuta*, a protective effect has been demonstrated against colitis in the affected host [23]. However, the relative contribution of immune cells in mitigating the Th1-dominated inflammatory response during helminth-mediated suppression of gut inflammatory diseases is unclear. Given that MCs modulate Th2 responses during *H. diminuta* infection, it was of interest to determine whether these cells contribute to protection against dinitrobenzene sulfonic acid (DNBS) induced colitis in *H. diminuta* infected mice. DNBS has been used as an effective agent to recapitulate colitis *in vivo* [38]. Using this DNBS-induced colitis model, it was observed that both MC deficient and WT mice infected with *H. diminuta* maintained similar heightened levels of protection against colitis compared with uninfected mice, suggesting that MCs do not play any major role in this protection. Other studies have demonstrated that the epithelial-derived cytokine IL-25 mediates the anti-inflammatory protection by *H. diminuta* in DNBS-induced colitis [39]. However, it is likely that the protection against colitis exhibited by *H. diminuta* and partly mediated by IL-25 occurs independent of MC. Other factors might include an involvement of regulatory T cells and/or other myeloid cells involved in promoting a Th2 response caused by the presence of the worm. These and other possibilities provide exciting areas for additional research.

In conclusion, the study by González et al. [24] has increased our understanding of the host cellular factors involved in immune responses against *H. diminuta*. Results from the present study demonstrate that MCs do contribute to the timely expulsion of *H. diminuta*. Further, MC-deficient animals display an altered cytokine expression kinetic profile resulting in a delayed expulsion of intestinal helminths. The authors also suggest that MCs are involved in the basal expression of IL-25, IL-33 and TSLP by epithelial cells (Figure 1). A key question remains regarding the degree of MC depletion in *Kit^W-sh/W-sh^* mice during *H. diminuta* infection and what subset of MCs (mucosal MCs and/or connective tissue MCs) are depleted in this model. Nevertheless, it is clear that MC mediated immunoregulation during helminth infection is of great interest, given that the strong Th2 immune response generated during infection by helminths has been linked to a positive prognosis or shown to have a beneficial effect in many autoimmune and neoplastic diseases [40–42]. Consequently, therapeutic applications developed as a result of an increased understanding of helminth-associated immunomodulation, as well as the involvement of MCs in response to helminth infection, remains an appealing and worthwhile goal.

**Figure 1:**
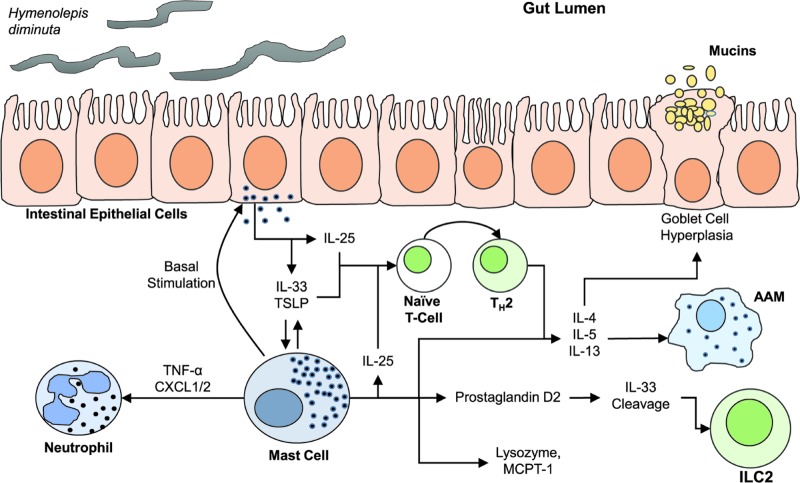
Proposed mechanism for the action of mast cells during the immunological response to helminth infection by *Hymenolepis diminuta* Mast cells stimulate intestinal epithelial cells causing a constitutive expression of basal IL-25, IL-33 and TSLP. The presence of these cytokines in the intestinal lumen is crucial to an efficient immune response required for timely expulsion of helminths. Detection of helminths by the epithelial cells cause an increased release of cytokines IL-25, IL-33 and TSLP, resulting in the activation of mast cells and other Th2 lymphoid and myeloid progenitors. Mast cells secrete a wide variety of cytokines and growth factors including IL-4, IL-5, IL-13, IL-25, IL-33, TNF-α, CXCL1, CXCL2, and TSLP, MCPT-1, prostaglandin D2, and lysozyme. CXCL1, CXCL2 and TNF-α activates neutrophils, prostaglandin D2 production activates type 2 innate lymphoid cells, while IL-4, IL-5 and IL-13 activates alternatively activated macrophages. Mast cell derived IL-25 stimulates the Th2 immune response. Further, mast cell degranulation results in anti-helminthic effector mechanisms including goblet cell hyperplasia, increased mucin production, smooth muscle contraction and increased peristalsis, leading to helminth expulsion.

## Abbreviations

AAI: allergic airway inflammation
CIA: collagen-induced arthritis
DIO: diet-induced obesity
DNBS: dinitrobenzene sulfonic acid
DSS: dextran sulfate sodium
EAE: experimental autoimmune encephalomyelitis
IL: interleukin
MC: mast cell
MCPT-1: mast cell protease 1
MLDS: multiple low-dose streptozotocin-induced diabetes
NOD: non-obese diabetic
OVA: ovalbumin
TNBS: trinitrobenzene sulfonic acid
TNF: tumor necrosis factor
TSHR: thyroid stimulating hormone receptor
TSLP: thymic stromal lymphopoietin
VEGF: vascular endothelial growth factor
